# Effect of the Presence of Nuchal Cords on Vaginal Breech Labor

**DOI:** 10.7759/cureus.39769

**Published:** 2023-05-31

**Authors:** Shunji Suzuki

**Affiliations:** 1 Obstetrics and Gynecology, Japanese Red Cross Katsushika Maternity Hospital, Tokyo, JPN

**Keywords:** fetal heart rate, perinatal outcomes, vaginal labor, nuchal cords, breech presentation

## Abstract

Objective: Breech delivery has been reported to be associated with a high incidence of abnormal fetal heart rate pattern leading to neonatal asphyxia. In this study, we examined the effect of the presence of nuchal cords on perinatal outcomes of vaginal breech labor.

Methods: We reviewed the obstetric records of all singleton breech labor cases at the Japanese Red Cross Katsushika Maternity Hospital between 1999 and 2011. Of the 266 breech labor cases in singleton pregnancies, the presence of nuchal cords was recognized in 50 cases (18.8%) on neonatal findings at birth. We compared the clinical characteristics and perinatal outcomes between the breech labor cases with and without nuchal cords. A chi-square test was used for categorical data, and a p-value <0.05 was considered significant.

Results: It was found that the incidence of fetal heart rate abnormality during breech labor or neonatal asphyxia in the deliveries with nuchal cords was not significantly different from that in those without nuchal cords. Also, there was no significant difference in the rate of emergent caesarean delivery between the two labors with and without nuchal cords.

Conclusion: The current results suggest that the presence of nuchal cords may not be associated with perinatal outcomes. Our results may become one of several pieces of evidence leading to the alleviation of anxiety in pregnant women with breech presentation and fetal nuchal cords.

## Introduction

A nuchal cord occurs if the umbilical cord gets wrapped around the fetal neck. The presence of nuchal cords seems common, and while they usually do not cause any adverse outcomes, in rare cases, serious complications can occur. The effect of nuchal cords on the outcome of vaginal delivery may be controversial [1]. In most studies with singleton deliveries, nuchal cords have been observed to be common and rarely associated with adverse outcomes in neonates [1-4]. Although in the deliveries of singleton presentation with nuchal cords, fetal bradycardia and/or variable decelerations in the fetal heart rate may occur more than those without nuchal cords, no significant difference has been observed in the rate of operative deliveries or in the incidence of neonatal asphyxia between the two deliveries [1-4].

To date, breech delivery has been reported to be associated with a high incidence of abnormal fetal heart rate pattern leading to neonatal asphyxia [5]. However, there have been a few investigations in the perinatal outcomes of vaginal breech delivery, except for the cases of the second twin after vaginal delivery of the first twin [6]. In this study, we examined the effect of nuchal cords on vaginal breech labor retrospectively.

## Materials and methods

This was a retrospective study of singleton pregnancies managed at the Japanese Red Cross Katsushika Maternity Hospital between 1999 and 2011. This period was chosen because in the last decade, most breech presentations were delivered by an elective caesarean section [7]. During the period, there were 22,622 deliveries at ≥22 weeks of gestation managed at our institute. The protocol for the current study was approved by the Ethics Committee of the Japanese Red Cross Katsushika Maternity Hospital (K2021-30).

In the current study, we reviewed the obstetric records of all singleton breech deliveries. Demographic information and the characteristics of labor were extracted from patient charts. Of 22,622 deliveries, there were 1,064 deliveries with singleton breech presentation without abnormal placental position (4.7%). The indications for trial of breech vaginal labor at our institute are as follows: (1) maternal request for vaginal delivery of breech presentation under informed consent regarding the risk of vaginal breech labor, (2) estimated fetal weight of 2,500-3,800 g, and (3) universal flexion attitude (a position of the fetus where the fetal chin is in close contact with the chest; denial of a hyperextended neck by ultrasonography). The presence or absence of nuchal cords was not considered in the condition for vaginal delivery.

Partial breech extraction of the lower (or the remaining) part of the fetal body was performed with the technique called ‘transverse figure 8 breech delivery (TF8 maneuver)’ [8]. In Japan, the procedure has been widely recommended for reducing the incidence of a nuchal arm. In the procedure, the fetal body must be pulled while twisting two or three times as in drawing the transverse number 8 in a direction until the upper limbs are delivered.

Of the 1,064 breech deliveries, 266 (25.0%) met eligibility criteria and underwent the trial of vaginal labor. Of the 266 pregnancies, the presence of nuchal cords was recognized in 50 cases (18.8%) on neonatal findings at birth. The number of cases of neck coiling of the umbilical cord was 42 cases once (84%) and 8 cases more than twice (16%). In the current study, we compared the clinical characteristics and perinatal outcomes such as the rate of emergent caesarean delivery, fetal heart rate abnormality during labor and neonatal asphyxia between the breech labors with and without nuchal cords.

Data are presented as number (percentage). Statistical software SAS, version 8.02 (SAS Institute Inc., Cary, NC) was used for statistical analyses. A chi-square test was used for categorical data, and a p-value <0.05 was considered significant.

## Results

The clinical characteristics of women with singleton pregnancies of breech presentation who met the eligibility criteria and underwent trial of labor are shown in Table 1, while Table 2 shows the perinatal outcomes. There were no significant differences in the clinical characteristics such as parity, maternal age, type of breech presentation and neonatal birth weight between the deliveries with breech presentation with and without nuchal cords. The incidence of fetal heart rate abnormality during labor or neonatal asphyxia in the deliveries with nuchal cords was not significantly different from that in those without nuchal cords as shown in Table 2 (p = 0.07, 0.57, respectively). In addition, there was no significant difference in the rate of emergent caesarean deliveries between the two labors with and without nuchal cords (p = 0.65), regardless of the attributes.

**Table 1:** Clinical characteristics of breech labor cases in with and without nuchal cords Data are presented as number (%).

	Nuchal cords		p-value
	Absent	Present	
Total number	216	50	
Nulliparity	111 (51)	26 (52)	0.94
Maternal age ≥35 years	44 (20)	15 (30)	0.48
Simple breech presentation	147 (68)	32 (64)	0.58
Neonatal birth weight ≥3,500 g	8 (4)	2 (4)	0.92

**Table 2:** Perinatal outcomes in breech labor cases with and without nuchal cords Data are presented as number (%). *Fetal heart rate (FHR) abnormality: fetal bradycardia and/or variable decelerations in the fetal heart rate

	Nuchal cords		p-value
	Absent	Present	
FHR abnormality*	124 (57)	21 (42)	0.07
Emergent caesarean delivery	127 (59)	27 (54)	0.65
Nulliparity	86 (68)	16 (59)	0.39
Maternal age ≥35 years	29 (23)	8 (30)	0.45
Simple breech presentation	80 (63)	14 (52)	0.28
Neonatal birth weight ≥3,500 g	2 (2)	0 (0)	0.51
Neonatal Apgar score <4 at 1 min	11 (5)	1 (2)	0.57
Neonatal Apgar score <7 at 5 min	0 (0)	0 (0)	1
Umbilical artery pH <7.1	4 (2)	1 (2)	0.94
Umbilical artery pH <7.0	0 (0)	0 (0)	1

## Discussion

In this study, the incidence of nuchal cords in cases of trial of breech labor (18.8%) was almost similar to that reported previously [1-4]. However, the presence of nuchal cords in breech presentation did not seem to be associated with the fetal heart rate abnormality or adverse perinatal outcomes in cases of vaginal breech labor. The current results suggest that the presence of nuchal cords may not be of lesser concern in cases of breech vaginal labor than those of vertex labor.

The most common complication of nuchal cords has been reported to be the decreased fetal heart rate during delivery [1-4]. However, in the current study, with breech labor, there were no statistical differences in the fetal heart rate abnormality between labor cases with and without nuchal cords. During labor, the umbilical cord can become compressed during uterine contractions. In cases of labor with breech presentation, cord compression susceptibility may not be higher than in those with vertex presentation because the umbilical cord is pulled out of the uterus at a shorter distance. Furthermore, the presence of umbilical cord prolapse has been reported to be the most serious condition associated with adverse outcomes in breech labor requiring an emergency caesarean section [9,10]. However, the condition may be less likely to occur in breech presentation with nuchal cords because the umbilical cord is usually located above the uterine cavity. We believe that the current results will support these hypotheses. In addition, it seems to be common for pregnant women to become anxious when ultrasonography reveals the presence of nuchal cords (the presence of umbilical cord coiling around the fetal neck at least once) [11,12]. Our study results will hopefully become one of the several pieces of evidence leading to the alleviation of their anxiety.

We understand the presence of some serious limitations in the present study including a small sample size and a high rate of emergent caesarean deliveries. First, in this study we did not examine the types of nuchal cords. For example, Type A nuchal cords are free-moving and may come undone naturally, while Type B nuchal cords do not [13]. Nuchal cords have been observed to occur in more than one in four births; however, the incidence of Type B nuchal cords has been observed to be in 1 in 50 deliveries. Emergency caesarean section and stillbirth have been reported to be associated with Type B nuchal cords [13]. To our knowledge, there seem to be few studies on breech labor concerning the difference between types of breech presentation. Therefore, types of breech presentation must be recognized at vaginal delivery. Second, we did not examine the length of the umbilical cord; short or long umbilical cords may affect multiple nuchal cords or true umbilical knots during labor at near term [14]. Therefore, a further large study examining the condition of the umbilical cord may be needed to assess the effect of nuchal cords.

## Conclusions

Our study results suggest that the presence of nuchal cords may not be associated with the perinatal outcomes; therefore, we may not need to pay attention to the presence of nuchal cords in cases of breech vaginal labor. In addition, the results may become one of the several pieces of evidence leading to the alleviation of anxiety in pregnant women with breech presentation and fetal nuchal cords.
